# How to Promote the Development of Nurses’ Moral Competence According to Patient Representatives: A European Qualitative Study

**DOI:** 10.1155/jonm/6655643

**Published:** 2025-12-11

**Authors:** Alessandro Galazzi, Minna Stolt, Evridiki Papastavrou, Nikos Stefanopoulos, Maria Dimitriadou, Johanna Wiisak, Brian Keogh, Stefania Chiappinotto, Catherine McCabe, Riitta Suhonen, Chris Gastmans, Alvisa Palese, the PROMOCOM Study Group

**Affiliations:** ^1^ Department of Medicine, University of Udine, Udine, Italy, uniud.it; ^2^ Department of Nursing Science, University of Turku, Turku, Finland, utu.fi; ^3^ Wellbeing Services County of Satakunta, Pori, Finland; ^4^ Department of Nursing, School of Health Sciences, Cyprus University of Technology, Limassol, Cyprus, cut.ac.cy; ^5^ Cyprus Nurses and Midwives Association, Nicosia, Cyprus; ^6^ Department of Nursing, University of Patras, Patras, Greece, upatras.gr; ^7^ School of Nursing and Midwifery, Trinity College Dublin, Dublin, Ireland, tcd.ie; ^8^ Wellbeing Services County of Southwest Finland, Turku University Hospital, Turku, Finland, vsshp.fi; ^9^ Department of Public Health and Primary Care, KU Leuven, Leuven, Belgium, kuleuven.be

**Keywords:** focus groups, moral competence, nurses, nursing, patient involvement

## Abstract

**Aim:**

To explore (a) how nurses can be supported in developing their moral competence and (b) which tools should be used, according to the perspective of patient representatives.

**Background:**

Nurses must provide morally competent care. Examining how moral competence can be developed requires a comprehensive approach that includes patients and their representatives to complement the perspective of professionals (e.g., educators).

**Methods:**

A descriptive qualitative multinational study in 2023 as part of the Promoting a Morally Competent Nurse (PROMOCON) project. A purposive sample of 35 patient representatives as members or chairs of patient organisations representing different diseases or health issues in Belgium, Cyprus, Finland, Greece, Ireland and Italy. Data were collected through focus group interviews and analysed using a thematic and content analysis approach. The checklist of the Consolidated Criteria for Reporting Qualitative Research was followed.

**Results:**

The development of nurses’ moral competence can be supported at three different levels, namely (1) at the societal level: “Recognition of the nursing profession”; (2) at the organisational level: “Building organisations based on ethically sound premises”; and (3) at the level of individual relationships: “Working on oneself to achieve moral competence in relation to oneself and others”. To support the development of morally competent nurses, various educational measures, infrastructures and social tools were recommended.

**Conclusion:**

In this area, a systematic approach is required that includes multicomponent strategies and is complemented by practical tools.

**Implications for Nursing Management:**

This is the first study to address the question of how to support the development of a morally competent nurse from the perspective of patients’ representatives. The list of strategies and tools that have emerged can be considered by societies, professional organisations and academic institutions as well as national health services and the private sector to take concrete action.

## 1. Introduction

Nurses are expected to possess theoretical knowledge and technical skills, but also ethical competence, guiding them in making morally based decisions and respecting the values and rights of patients to provide patient‐centred care [1]. Moral competence is overall defined as comprising ethical sensitivity, knowledge, reflection, decision‐making, action and behaviour [2] and is underpinned by theoretical (e.g., values and norms) and practical (e.g., problems) aspects of ethics.

In daily practice, nurses undertake multiple activities with ethical implications [3], such as managing the confidentiality of information, taking and discussing ethically sensitive decisions with other colleagues, and respecting the choices of their patients. All nursing practice and interactions are intrinsically guided by moral competence [3]. However, although several studies have described how to support the development of nurses’ moral competence [4], to our best knowledge none has considered the perspective of patients or that of patient representatives. Enriching the available knowledge by including nonprofessional views may provide additional educational, clinical, managerial and research insights to design strategies aimed at preparing morally competent nurses.

Different strategies have been documented as effective in developing moral competence among nurses. In undergraduate education, students are expected to be exposed to positive role models and learning experiences [5] consisting of virtues, values and critical reflection [2]. The learning environment may provide opportunities for students to perceive and experience the link between ethics, professionalism and nursing practice [6]. During their professional development, nurses may further advance their moral competence after being exposed to human (i.e., individual, social, managerial and professional) and structural strategies (i.e., educational, environmental, organisational and societal) [4]. However, evidence provided by 29 studies published between 2007 and 2022 has involved nurses, nursing students or a mix of healthcare professionals [4]: despite reports of nurses’ moral competence affecting patient care [2], no studies have involved patients’ views on how such competence should be developed.

Trust between nurses and patients are based on professional competence and ethical behaviour on the part of nurses, who must demonstrate respect, integrity and empathy towards patients to establish an effective therapeutic relationship [7]. While receiving nursing care, patients and their families may experience both negative and positive impacts as the result of different levels of moral competence possessed by nurses. Patients may perceive high or low moral competence of nurses while witnessing their work processes and interactions in the care environment or when nurses share their difficulties with patients [8]. Therefore, while nurses have the privilege, like all professions, of establishing the fundamental requisites to develop moral competences [9], patients and their families should be considered among the stakeholders, since it is they who find themselves directly influenced by nurses’ moral competence in the healthcare context [10]. Valuing patients’ experiences, perceptions and views in the patient‐reported experience (PRE) and outcome(s) (PROMs) frameworks [11] may complement the evidence produced from the side of professionals. Moreover, all discourses regarding moral competence impose strategies limiting and/or preventing power imbalances between professionals (researchers included) and patients [12, 13]. Investigating how to develop moral competence requires an inclusive approach, involving a range of people with different perspectives [14]. However, despite these premises, to our best knowledge no studies have been conducted to date from the point of view of patients and patients’ representatives on the topic of how the moral competence of nurses can be developed.

Therefore, the aims of the study were to explore from the perspective of patient representatives (a) how nurses can be supported in developing their moral competence (e.g., knowledges, skills, attitudes and performances) and (b) which tool should be used. Two research questions were pursued: (a) What strategies can support nurses in developing their moral competence? (b) According to patients’ representatives, what tools can be used in such support?

## 2. Methods

### 2.1. Design

A qualitative descriptive multinational study using focus group interviews [15] was conducted in 2023 within a large project named the Promoting a Morally Competent Nurse (PROMOCON) project, an Erasmus + project to foster an educational programme for the development of morally competent nurses [16]. The intention of the large project was to develop a Massive Online Open Course to support the development of moral competence. In the first phase of the needs analysis, the literature was reviewed [4], and the perspectives of various stakeholders [17, 18] were taken to gain insights into what constitutes a morally competent nurse. The focus group was selected as the best method due to its capacity to generate unique data based on the synergy of the group interaction [19]. The Consolidated Criteria for Reporting Qualitative Research (COREQ) checklist [20] was followed (Supporting Table 1).

### 2.2. Participants

Purposive sampling [15] was employed to identify the most relevant and knowledgeable participants. Focus groups composed of between four (minimum) and nine (maximum) participants per partner country (Belgium, Cyprus, Finland, Greece, Ireland and Italy) were expected, amounting to between 24 and 54 participants in total, as suggested in the literature when sensitive issues are explored [21].

As members of accessible patient associations, eligible patient representatives were identified by country partners. To protect from intrusive and unnecessary interviews, those representatives being cared for in healthcare organisations, with ongoing processes of care, were all excluded.

Participants were invited by researchers in each country via telephone or email. An information brochure was provided, explaining the purpose of the focus group study and the PROMOCON project in general, the role and rights of the participants, and contact details of the research team. All those contacted accepted the invitation.

### 2.3. Data Collection

Focus group interviews were designed according to the guide developed by the Finnish team (Supporting Table 2) and piloted by the Italian team with two participants prior to the official initiation of the focus groups.

At least two researchers for each country were involved as the moderator conducting the interview and the observer collecting notes during the interview. The native language was used. Participants were invited to sit in a circle to facilitate the discussion, in a calm environment, with no visual or acoustic distractions, on the date proposed and agreed upon by all. Audio recording of the interview was ensured after receiving informed consent; moreover, notes were also collected in the field. The focus groups were conducted by expert researchers with different backgrounds (nursing, bioethics; see authors). The aims of the focus groups were shared at the beginning of the focus groups; having addressed doubts and questions, the interview began. No other persons were present during the data collection. The interview guide (Supporting Table 2), including also the collection of some sociodemographic data, was followed by all countries. The duration of interviews was approximately 2 hours; there was no break, as the participants indicated that it was not necessary. No repeated interviews were conducted.

### 2.4. Data Transfer, Analysis and Rigour

All focus group discussions in each country were transcribed verbatim in the respective national language. A thematic analysis [22] and a content analysis [23] were then carried out, initially in the native language.

Based on the narratives, to answer the first research question—*What strategies can support nurses in developing their moral competence?*—a list of codes with meaningful quotes from two members in each country was created and reviewed by a third member if questions or discrepancies arose. Each code and the meaningful quotes were translated into English in collaboration with English‐speaking experts and sent to the PROMOCON team for initial assessment. The overall data was then generally analysed in a cross‐country meeting to maintain the quality of the process.

The data was then analysed by a subgroup of researchers at a cross‐national level, with representation from all partner countries, to ensure the rigour and accuracy of the coding process. The codes were compared for similarities and differences, and, where present, subthemes were generated. The most representative quotes were selected, indicating their origin: Belgium (BE), Cyprus (CY), Finland (FI), Greece (GR), Ireland (IR) or Italy (IT). Not all themes generated subthemes; moreover, themes and subthemes were then categorised into levels as suggested by available literature [4, 24].

For the second research question—*What tools do patient representatives, believe can be used to provide such support?*—the content analysis approach [23] was used, and the data was inductively analysed, to identify and describe tools. Two researchers at the country level identified the first list of codes (first independently and then in agreement); disagreements were resolved by consensus with a third researcher. The codes were then translated and compared at the international level to check for similarities and differences. The final list was approved in an international meeting. One author (CG) supervised the entire process, while supervisors were appointed at each country level (see the authors).

The rigour and trustworthiness of the study were enhanced by the sustained commitment and reflexivity of the team, which ensured deep understanding and involvement in the process from data collection to analysis [25]. Reliability was also promoted by the study protocol, which was based on international scientific guidelines [26] and strictly adhered to by all countries. The Trail Code was created at the national level (available from the authors) with meaningful codes and quotes and then reviewed at the transnational level in a meeting. The credibility [25] of the findings was also ensured by (a) several online and face‐to‐face meetings of the subgroup involved in the data analysis and the whole research team to promote reflexivity and (b) the triangulation of the data analysed first at the national and then at the transnational level. Finally, the transferability of the results was promoted by the involvement of patient representatives on various healthcare issues and by checking the saturation level of each theme [27] by the entire research team at intra‐ and transnational levels (see authors).

### 2.5. Ethical Considerations

The study was approved by an ethical committee (EC) or institutional review board (IRB) in Cyprus, EC 2023.01.82; Greece, EC 29192/11‐4‐23; Ireland, EC 230501; and Italy, IRB 33/2023. According to Belgian and Finnish regulation, ethical approval was not necessary given the nature of the study, but permission was requested from the university departments where the study took place.

Participation in the study was voluntary; the written informed consent was provided in a form ensuring that participants were free to withdraw from the study at any time. The interviews were audio‐recorded with participants’ permission. Anonymity in reporting was ensured by using an identification number, avoiding any personal details of participants. To reflect the provenience, the country was reported in the quotes when presented (e.g., Finland [FI]). Each participant’s privacy and confidentiality were strictly maintained, and the focus group recordings were secured. The signed consent forms were locked in the archives of the university departments and filed separately from any other study materials that might link data to participants.

## 3. Results

### 3.1. Characteristics of Participants

A total of 35 patient representatives were involved, mostly females (*n* = 24, 68.6%) and aged between 40 and 59 years (*n* = 20; 57.2%). They represented associations concerning patients’ rights (*n* = 7, 20%), older people (*n* = 6, 17.1%), and mental healthcare (*n* = 4, 11.4%) as members (*n* = 26, 74.3%) or presidents (*n* = 9, 25.7%) of the associations. Twenty‐eight (80%) had experienced hospital admission(s) in their lifetime, as summarised in Table 1.

**Table 1:** Characteristics of participants stratified by countries.

Characteristics	Description	Countries						
		Belgium	Cyprus	Finland	Greece	Ireland	Italy	Total (%)
Participants	Number	9	5	5	6	4	6	35 (100)

Gender	Female	6	2	3	5	2	6	24 (68.6)

Age, years	20–29	1	0	0	2	0	0	3 (8.6)
	30–39	2	1	0	0	1	3	7 (20.0)
	40–49	0	1	2	2	2	1	8 (22.9)
	50–59	3	2	2	2	1	2	12 (34.3)
	60–69	1	0	1	0	0	0	2 (5.7)
	≥ 70	2	1	0	0	0	0	3 (8.6)

Highest education level	University education	8	5	4	6	4	3	30 (85.7)
	Secondary school	1	0	1	0	0	3	5 (14.3)

Association, for	Patients’ rights	0	0	2	1	2	2	7 (20.0)
	Older people	0	4	0	1	1	0	6 (17.1)
	Mental health	3	0	0	0	1	0	4 (11.4)
	Neurological diseases	3	0	2	1	0	0	6 (17.1)
	Oncological diseases	1	0	0	1	0	4	6 (17.1)
	Palliative care	1	0	0	1	0	0	2 (5.7)
	Bioethics	0	1	0	0	0	0	1 (2.9)
	Chronic obstructive pulmonary disease	1	0	0	0	0	0	1 (2.9)
	Heart diseases	0	0	1	0	0	0	1 (2.9)
	Infectious diseases	0	0	0	1	0	0	1 (2.9)

Role in the association	Chair/President	2	3	0	3	0	1	9 (25.7)
	Member	7	2	5	3	4	5	26 (74.3)

Hospitalisation experience(s)	Yes	8	5	2	6	4	3	28 (80.0)

### 3.2. Strategies to Support the Development of Moral Competence

The patient representatives propose a complex bundle of strategies that are articulated at a societal, organisational and individual level. At the societal level, they emphasise the need for “Recognition of the nursing profession”, at the organisational level, the need to “Build an organisation based on ethically sound premises”, while at the individual level they suggest that everyone should “Work to develop moral competence in relation to themselves and others” (Table 2).

**Table 2:** Supporting the development of nurse’s moral competence: levels, themes, subthemes and codes.

Level	Themes	Sub‐themes	Codes
Societal	Recognising the nursing profession	Ensuring media visibility and a positive societal narrative	Highlighting achievements
			Raising awareness regarding nurses’ role
		Acting for ethically sustainable working conditions	Ensuring resources and rules for acceptable working conditions
			Exchanging good practices nationally

Organisational	Building organisations based on ethically sound prerequisites	Being close to nurses	Reducing distances and softening hierarchies
			Facilitating closeness
		Ensuring occasions to debate	Promoting formal (cyclical and regular) and informal occasions for discussion
			Providing space and role for clinical nurses on ethics committees
		Promoting ethically sustainable working conditions	Promoting adequate staffing
			Managing effective deployment strategies across settings
			Leaders acting as role models/good managers
			Promoting psychologically safe working environment

Individual‐relational	Working on oneself towards moral competence in relation to oneself and others	Being prepared on ethical issues	Being educated
			Being a reflective practitioner
		Being open to others	Listening to others
			Communicating constructively
			Asking for/offering feedback
			Following and serving as good examples
		Making an ethically oriented community	Challenging colleagues in a positive way
			Valuing the patient perspective
			Allowing nurses to take on professional responsibility
			Identifying points for development

#### 3.2.1. Societal Level

At the societal level (Table 2), patient representatives called for recognition of the nursing profession: conceptually, recognition of the nursing profession refers to the broad societal recognition and appreciation of the fundamental contribution that nurses make to health systems and the overall well‐being of communities. It is a dynamic cultural and structural process aimed at enhancing the visibility, dignity, and empowerment of nurses at all levels of society. First, outside the healthcare system and concerning citizens, patient representatives believed that societies must devote spaces in the media to ensuring visibility and a positive narration of nurses’ role, where achievements are highlighted and negative information or examples of poor care are avoided or not emphasised:

We demand a lot from the hospitals and nurses, but you also hear positive opinions, and acknowledgements associated with their practices from the media, and we [citizens] understand the role and tasks in nurses’ professional performance (CY).

Moreover, patient representatives suggested the importance of raising awareness within society of the complexity of nurses’ role in providing care by informing citizens and helping them to understand why daily failures may occur. Rather than claiming more attention, putting nurses in a more difficult position or exposing them to aggressive behaviours, raising awareness may increase empathetic behaviours among citizens:

Maybe if people knew about the role of nurses, they would be more empathetic and understanding in difficult times (GR).

Secondly, patient representatives asked for action internally by the National Health Service to provide resources for sustainable working conditions. They believe that the entire society is responsible for the working conditions where nurses are placed, given that resources and priorities are decided at the political level: they should be adequate to prevent excessive stress, pressure and difficulties that may trigger burnout, reduced nursing care and, ultimately, moral distress. Therefore, rendering working conditions acceptable may help the nursing profession to thrive simply because, under these conditions, nurses can fully express themselves and their potential.

[It is about] being recognised and seen for one’s attitudes, having the possibility of developing them and then being placed in a context where a person can express the best of himself/herself, giving satisfaction to others, because, in any case, ours is a helping profession, and we are successful when people feel better (IT).

Moreover, detecting and disseminating good examples of practice at the national level, rendering nursing care more visible, may motivate the efforts of the society, ultimately leading to “visible strategies to meet patients’ needs” (IR).

Sharing examples is a clear demonstration that it is possible to do better, especially for those healthcare settings experiencing a difficult situation.

#### 3.2.2. Organisational Level

According to the patient representatives, healthcare facilities should “build their organisations on the basis of ethically sound prerequisites” (Table 3). Ethically sound organizational foundations in healthcare refer to the structural and value‐based principles according to which healthcare facilities are designed and operated, and which ensure that care is provided with respect for human dignity, professional integrity, and democratic participation.

**Table 3:** Tools suggested to support the development of nurse’s moral competence.

Tools	Description
Education	*Undergraduate nursing education*
	Recruiting teachers:
	‐ Inspire teachers
	‐ Know ethics theories, values and principles of legislation
	‐ Have practical experience
	Ensuring that teachers use effective teaching strategies
	‐ Simulate scenarios regarding ethics
	‐ Real‐life cases
	Exercising ethical thinking in all education settings:
	‐ Classroom
	‐ Clinical learning environment and supervision
	Evaluating competences with validated tools
	*Continuing education*
	Requiring mandatory education on ethics throughout the professional life
	Designing and offering:
	‐ Interdisciplinary education
	‐ Practical‐based education
	Considering communicational and time management competences as central

Infrastructure	Ensuring ethics consultation and reflection
	Offering workplace counselling in case of issues (e.g., burnout)

Policies	Designing and implementing patient‐centred policies/guidelines

While designing the organisation’s structure, those responsible should create the preconditions to ensure continuous awareness of the intrinsic ethical nature of the nursing profession. Organisations structures should be designed to “be close to nurses”, expressing support for them by integrating nurses and all healthcare professionals, from the bedside to the managerial level. This means that the organisation should be vertically integrated, overcoming the frequent disaggregation and distance between managerial responsibilities and clinicians.

The managers often have so little contact with the workplace… the management or the care manager supports a certain decision or protocol—e.g., euthanasia is allowed in this hospital, or how we deal with advance directives… (BE).

By softening the hierarchies, the nurses can feel supported, openly communicate and share challenging situations that may occur in daily practice. In this context, nurses are encouraged to express feedback and possible solutions to nurse managers, who have the ability to change and to undertake organisational decisions, thus valuing nurses’ opinions and ultimately empowering them.

It is often one‐way traffic, like “We say this is how it should be done, and you just have to do it without saying that this is not feasible in the workplace, or it could be done differently…”. So two‐way is very important (BE).

These organisations implicitly coach nurses in developing their moral competence: conversely, those healthcare services strongly based on hierarchical structures, distancing managerial positions from those at the bedside, prevent open discussion and debate regarding sensitive issues.

Some nurses have only worked in hierarchical systems and don’t know how to be part of a team (IR).

Organisations should also “provide concrete occasions to debate” the ethical issues encountered by nurses in their practice on both informal (during coffee breaks) and formally designed occasions, where they are called to present cases and to ask for or offer a point of view.

[This keeps] issues on the surface, raising them for discussion on a cyclical and regular basis (FI).

Nurses should be involved on ethics committees to contribute and address issues, also serving as a reference point for other nurses, “handling feedback, what actions to take, policies for practice” (FI).

Nurses should be recognised for the time spent on ethical discussions, avoiding involving them in their free time; moreover, they should be formally invited in the meetings, giving full visibility of their point of view as part of the priorities of the organisation, an aspect often neglected.

Nurses in some areas don’t argue back or give an opinion, and this is a problem because of hospital and nursing management not being supportive (IR).

Patient representatives recommend the provision of “sustainable working conditions” by ensuring adequate staff, which is doubly connected with ethical issues: on the one hand, nurses may not feel respected if they are left in poor staffing conditions for a long time in terms of nurses available; on the other hand, they have to ration care when there are few of them, thus missing some interventions required by patients.

I believe that understaffing and working conditions affect the ethical values of nurses … a fact that brings the nurses to certain limits that conflict with ethical values on various issues (CY).

Alongside an adequate number of nurses, sustainable staffing also concerns the competence of nurses to ensure the care required in each specific care setting. When deployment is made from one setting to another under urgent conditions and nurses without competences are moved to care for other patients, they may feel unsupported.

So surely making the recruitment mechanisms, the transfer mechanisms, the shift mechanisms a little more respectful of the competence would also help (IT).

Patient representatives have also called for good nurse managers who should act as role models, showing effective ethical behaviours rather than merely indicating what is good or not.

[They should be] assertive enough to advocate for patients but not to tell another nurse that they did something wrong or unethical (IR).

All these strategies make a “psychologically safe environment” where nurses can freely express their own potentialities and limitations, not only inside the profession, among nurses, but also within the team, leading to “a psychologically safe working culture, [where] the work community is such that you dare to express your own limitations” (FI).

#### 3.2.3. Individual‐Relational Level

According to the patient representatives, one main theme emerged at the individual level: “working on oneself towards moral competence in relation to oneself and others” (Table 2). Conceptually, moral competence among nurses, as understood by patient representatives, refers to the continuous, self‐reflective development of individual ethical awareness, judgement and behaviour in relation to self and others in the healthcare environment. This includes a personal and interpersonal commitment to ethical growth as an integral part of professional identity and nursing practise.

Nurses should “be prepared” for how to manage ethical issues, a professional competence that must be learnt as an undergraduate and through continuing education opportunities. The capacity to be sensitive to ethical issues relies on reflection: each nurse should be prepared to be a reflective practitioner, capable of thinking critically both during nursing actions and after these actions in a comprehensive manner.

In a context where continuing ethical education is delivered in a multidisciplinary perspective, nurses can learn formal content but also exercise themselves in “being open”, listening to others and working with others, thus challenging and continuously growing in moral competence.

Providing nurses with as much interactive training as possible that corresponds to the reality of their profession in the course of their career and the exchange of experiences between individuals will help them to decide the best possible treatment of an incident (GR).

Moreover, ethical issues require discussion: thus, nurses should be prepared to establish “constructive communication” with the members of the team in order to detect ethical issues, anticipate them, discuss the underlying factors when they are encountered, and ask questions, challenging each other in a positive and constructive way.

Have constructive conversations with colleagues: reflect on each other’s actions and point out when something is not going well; ask the right questions and challenge each other in a positive way (BE).

Nurses should therefore be open to sharing both positive and negative experiences, where “critical reflections” can be sought or received by other members of the team, providing an external point of view, a strategy that can increase the moral competence of each individual nurse.

Yes, share experiences. And occasionally also play devil′s advocate and say: ‘But have you ever looked at it like this?’ (BE).

According to the patients’ representatives’ perspective, nurses should have and serve as a “good example”. Having exemplary leaders and peers may reinforce competences and continuously develop them, serving as an example, inspiring students or younger nurses by showing them how ethical issues may be managed like other clinical, technical or emotional issues.

They [students] look to their supervisors as role models for everything… and it is like “monkey see, monkey do”. If you see ethics in normal everyday practice, that is how you learn (IR).

To develop a “morally competent‐oriented community”, it has been suggested that nurses act as an example, spending time coaching new members—not only students, but also new graduate nurses or new colleagues.

Mentoring, work coaching [enables nurses] to see what it’s like to work within an ethically competent carer or within an ethically competent work community (FI).

In this community, each member is challenged in a positive way to do their best. Members are also invited to collect feedback from patients and ensure that their opinions are valued by all colleagues as part of the normal practice, not only when critical issues emerge but also in case of positive experiences that may encourage nurses to do their best.

Or also, if a patient says something about your colleague, you can go up to that colleague and say: ‘Hey, I heard that about you’, or ‘That patient said you were really nice this morning’ (BE).

Patient representatives suggested that nurses should be supported to undertake the full responsible of the nursing care, which implies everyday ethical decisions.

For me it is unethical that [nurses are avoided] to assume their responsibility for which they have been prepared (CY).

In this community, nurses may identify points for development in facing issues and overcoming internal (time restrictions) and external barriers (e.g., colleagues not ready to undertake an ethical conversation). By identifying points for development, nurses are supported to develop their competence continuously.

That is, it undoubtedly counts if the other person feels, how can I say, listened to. It is not just a number on a scoreboard, which does not mean precisely that one must always have a good time and the other never. So, you must be very careful even with those who deal with these things, and especially if there is a need to try to mediate, rather than letting discussions happen between people (IT).

### 3.3. Tools to Support the Development of Moral Competence

According to the patient representatives, education, organisational infrastructures and policies (Table 3) are tools useful to support the development of moral competence in nurses.

Ethics education should be designed and delivered in undergraduate education and then in continuing professional development courses. In basic education, the recruitment of teachers should be based on specific criteria: they should be inspiring, love teaching ethics, and use innovative teaching methodologies. They should know the ethics theories, ethical principles and legislation affecting nurses’ decision‐making processes, alongside solid practical competence in the clinical setting.

I think the most basic competencies are being knowledgeable on the subject that you teach, having work experience so that theory and practice combine, and being understandable to your students (GR).

Lessons should not only be theoretical: the use of simulated scenarios or cases regarding ethics involving patients is suggested, to learn through real‐life cases.

[It is not just about] telling the patient experience in training, that what happens there between the ears [patient], whether it would help some people to understand it again and behave in an ethical way (BE).

Students should be asked to exercise their ethical thinking in all education settings up to clinical rotations, where effective supervision should be provided to promote the development of complex competences, because ethics “is not just knowledge, it’s also doing” (BE).

Moreover, ethical competences gained during education should be assessed with validated tools to ensure formal feedback on the outcomes achieved.

In the continuing education, courses concerning ethical issues should be mandatory, offered to nurses and attended by them. Learning opportunities should be practical‐based and multiprofessional. “Education about ethical practice needs to be multidisciplinary/interprofessional and mandatory” (IR), thus capable of reflecting the real professional life of nurses with its dilemmas, concerns and issues that often require multidisciplinary approaches to exercise and refine their competences.

Moreover, participants recommended focusing continuing education on how to manage time, “how to efficiently use and control it to minimise burnout” (CY). Time management competence may increase the capacity to detect, discuss and take multiprofessional decisions to face ethical issues encountered in the professional life and ultimately to prevent the nurse’s moral distress. Furthermore, nurses should be continuously trained on communication strategies “that support in doing the best, […] with more peace of mind” (IT).

Organisational infrastructures are also addressed to help nurses when they need to make difficult decisions. There should be training for nurses to recognise their own suffering before failing in burnout, triggering depersonalisation, missed care and unintended violations of the right of the patients, and a counselling system on ethics should be offered.

It would be useful for nurses to learn ways to recognise when they are reaching their limits and when they get affected by burnout. Because when they reach their limits, it makes sense to have negative behaviours. So, through some training, they can recognise those points and signs that sound the alarm that something is going wrong, and you must work on it (CY).

Experts should be identified and appointed to offer a concrete service to all professionals. Nurses should be allowed in their working time to reflect and receive ethical consultation. Moreover, workplace counselling in case of issues (e.g., burnout) should be provided.

Social tools, such as policies aimed at emphasising and promoting patient‐centred values, safety, and models of care at the system level capable are suggested.

[There should be] a client and patient safety strategy and implementation plan so that there are consistent policies and feedback processes (FI).

Such policies make clear the quality of care expected, also providing appropriate resources. Indirectly, they express the support offered to nurses to ensure that the expected care is delivered.

## 4. Discussion

We investigated patient representatives’ perceptions of how nurses can be supported to develop their moral competence and what tools can be used to support them in developing such competence. Participants representing diverse service users (e.g., older people, people affected by mental disorders) in a European perspective provided insights that seem to be unique, given that no studies involving patient representatives have been performed to date [28].

### 4.1. Strategies to Support the Development of Moral Competence

Strategies have been categorised at the societal, organisational and individual‐relational levels, as proposed by Poikkeus et al. [24] and Wiisak et al. [4], who identified the micro‐, meso‐ and macrolevels to support nurses’ moral competence. These levels positively influence each other in the development of moral competence; however, while some reports at the individual and organisational levels may be traced in the available literature [29, 30], some new insights emerged.

The broadest level identified is that of the society, where actions to recognise the nursing professions move in two directions. Towards citizens, the society should promote positive visibility, capable of stimulating the credibility, dignity and value of the profession. Nurses may be recognised only by citizens who experience a health issue [31] and society as a whole often fails to recognise the importance of nursing work [32]; furthermore, the widespread stereotype of nurses [33] may negatively affect sensitivity towards them, preventing career attractiveness and a willingness to invest in the profession. Therefore, a society that promotes and preserves nurses’ professional dignity and value may support nurses in fully developing their profession, which is ethical by nature; a concrete promotion of the nursing profession may be seen when the society provides appropriate resources to national health services. In acceptable working conditions, nurses may feel respected, and this may allow their moral competences to flourish by, for example, prioritising patient‐centred care and improving their nursing practice [34]. Moreover, by disseminating examples of positive nursing care, society may be informed regarding nurses’ achievements; in turn, nurses may feel empowered by a society that shows respect and thus motivated to increase their moral competences.

Patient representatives reported strategies at the organisation level: this is the professional level where the nurses must be most supported in carrying out ethical practice in daily care, but it is also the one often missed [35]. Firstly, the interviewees suggested some prerequisites as the foundation of the organisation itself: nurses must be supported directly, softening hierarchies that may limit open discussion regarding ethical issues and increasing the feeling of being understood in terms of the complexity of daily care and its ethical nature. To date, perceptions of solitude and distance from managers have been linked with poor wellbeing, professional dissatisfaction and an intention to leave the profession [36], but not with the development of moral competence. From their side, nurse managers have been found to need to be close to their superiors to do their best [37]; meanwhile, staying close to nurses may help to develop their moral competence and perception of the ethical challenges that they experience in clinical environments [38], as nurse managers are currently mainly dealing with financial issues [39]. Organisations should also render the views of clinical nurses visible by involving them in ethical discussions, recognising their time in such participation and thus legitimating their need to discuss and their contribution to ethical issues. However, nurses are not members of ethical committees with active roles in all countries [40], and only recently has the value of clinical ethical meetings been underlined [41].

Providing appropriate staff shows respect for the nursing role: ensuring appropriate strategies to manage urgent understaffing, mapping competences and preventing the deployment of nurses from one unit to another without the appropriate competence reflect respect not only of nurses but also of patients [42]. Nurses who perceive sufficient job resources are likely to feel intrinsically motivated at work, and motivation is a source of professional development [43]. Moreover, nursing practice is challenged by organisational structures and the development of the healthcare system and may inhibit nurses’ decision‐making and compromise basic nursing values [44]. In this context, the role of nurse managers is important: they may inform policies regarding the adequacy of the staff [45], and they are in a position to see the impact of policies at the societal level [46, 47]. This occurs when nurse managers are close to nurses [48]: they can see the impact of decisions undertaken at the macrolevel and can act locally, negotiating more resources and acting as role models [49], thus advocating the nurses’ role. This suggests that the organisation should be internally coherent and open.

The patient representatives interviewed also indicated some strategies at the individual‐relational level as the personal responsibility of each nurse. To begin ethically competent personal growth, it is important to work on oneself, recognising the value of others’ opinions and of becoming members of a team. Awareness of the importance of one’s growth in moral competence is a personal aspect that requires professionals’ deep reflection from the beginning of their academic education and subsequently in their professional and private lives [50]. Some individuals are naturally inclined towards deep personal reflection, while others will need to be supported in developing the moral competence that requires certain attitudes, knowledge and skills. However, alongside the individual role of all professionals, they may compose an ethically sound community when their integration is promoted. This new strategy may help to develop moral competences due to its capacity to promote a sense of belonging, a perception of being appreciated and valued, and the possibility to share openly ethical issues that may, in turn, stimulate development of the competences possessed.

### 4.2. Tools to Support the Development of Moral Competence

Interviewees suggested practical tools to support the development of nurses’ moral competence. These should all be designed at the system level, as clear actions that may not only be useful for nurses. Education in ethics is considered mandatory in everyday and in professional life. However, to date, ethics has received limited consideration in universities, with no clear content and evaluation system [51]. Representatives of patients suggested clear requirements for teachers, who should be capable of inspiring and motivating students in ethical issues, possess knowledge and give practical examples, and act as role models every day, not just in extraordinary situations (e.g., ethical dilemmas) [44]. The same is true of the clinical supervision offered in practice, allowing students to see virtuous professional models. In other words, the interviewees recommend overcoming traditional educational approaches by introducing methods that facilitate the development of critical judgement and decision‐making abilities [6], where an accurate measure of the competence gained is ensured. Professional bodies in different countries that are responsible for regulating continuing education credits and recognition [52] should consider ethics education mandatory, while healthcare services, agencies and ethical committees should offer courses at different levels of complexity to satisfy nurses’ learning needs.

Healthcare facilities should provide internal or external services to all workplaces and nurses to support them in their reflection when ethical issues are encountered and when their wellbeing is compromised (e.g., burnout), given that in suboptimal conditions, their decisions may be compromised [53]. The infrastructure suggested by the patient representatives is in line with the multicomponent interventions already underlined by the literature to support nurses and prevent their moral distress [54]. Moreover, given that ethics is embodied in each nursing intervention at the bedside, policies that emphasise the value of patient‐centred care may function as supporting tools by influencing the values and the priorities of the healthcare system. The overarching system may situate the ethical discourse within the society, where values and priorities are set—thus external to the units where nurses work [55]. The macrolevel may inform decisions at the microlevel and may inform externally on the value of the nursing profession, the support that requires continuing interaction between social systems and the world of individuals as nurses or patients [56].

### 4.3. Strengths and Limitations

To our knowledge, the voice of patient representatives has never been raised when it comes to the development of moral competences in nurses. The study was international in scope and was conducted by a research group, qualified to conduct this research because of their experience in the field. However, the study has some limitations. Firstly, we included patient representatives to gather insights and perspectives at a European level. However, only some associations that are accessible at the local level were included; despite their diversity, certain perspectives may not have been included (e.g., palliative care, paediatrics), indicating the need for future studies. Secondly, the number of participants varied from country to country. Although the categories that emerged were screened for saturation, future studies could broaden participation and focus to other representatives (e.g., individuals presenting different vulnerabilities). Third, we used two different methods of data analysis, based on thematic and content analysis [22, 23]—according to the different aims underlying the research process. Although the combination of two methods is unusual, we employed strategies aimed at ensuring rigour. However, future studies using a similar multimethod analysis could confirm these findings. Fourth, some findings were not mutually exclusive—however, given the methodological decision to follow the framework recently established in field [4], and with the intention of leaving the findings at the most granular level, we did not combine them.

## 5. Conclusions

Several studies have provided suggestions and recommendations regarding how to develop a morally competent nurse. However, despite their relevance given the impact of nursing care on their lives, patients have not been involved to date in identifying strategies and tools to develop such competences. To cover the gap, focus groups involving patient representatives at the international level were conducted, resulting in a rich list of strategies targeting societal, organisational and individual‐relational levels. The overall findings suggest that a systematic approach, including multicomponent strategies complemented by practical tools, is required. The responsibility to support the development of morally competent nurses should be considered at all levels, by providing a continuous learning experience in an environment that can stimulate such development, in a context where nurses fulfil their responsibilities and societies value nursing care. The list of strategies and tools that emerged can be considered by societies, professional and scientific bodies, and national health services and private sectors to undertake concrete actions. Integrating these perspectives with those of other stakeholders (e.g., nurses) may be important to develop a comprehensive agenda of strategies.

## Disclosure

Each author should have participated sufficiently in the work to take public responsibility for appropriate portions of the content. All authors agreed to be accountable for all aspects of the work in ensuring that questions related to the accuracy or integrity of any part of the work are appropriately investigated and resolved. The European Commission’s support to produce this publication does not constitute an endorsement of the contents, which reflect the views only of the authors, and the Commission cannot be held responsible for any use which may be made of the information contained therein.

## Conflicts of Interest

The authors declare no conflicts of interest.

## Author Contributions

E.P., N.S., C.M., R.S., C.G., A.P.: made substantial contributions to conception and design; A.G., M.S., E.P., N.S., M.D., S.C., C.M., R.S., C.G., A.P.: acquisition of data or analysis and interpretation of data; A.G., M.S., E.P., N.S., M.D., B.K., S.C., C.M., R.S., C.G., A.P.: involved in drafting the manuscript or revising it critically for important intellectual content; A.G., M.S., E.P., N.S., C.M., R.S., C.G., S.C., A.P., J.W.: given final approval of the version to be published.

## Funding

This work was co‐funded by the Erasmus+ program of the European Union (agreement nr. 2022‐1‐IT02‐KA220‐HED‐000087544).

## Supporting Information

Supporting Table 1: COnsolidated criteria for REporting Qualitative research checklist.

Supplementary Table 2: Guide developed to conduct the focus group.

## Supporting information


**Supporting Information** Additional supporting information can be found online in the Supporting Information section.

## Data Availability

Data are available on request from the authors.
