# Supplementary material for: How to Promote the Development of Nurses’ Moral Competence According to Patient Representatives: A European Qualitative Study
Source: J Nurs Manag. 2025 Dec 11;2025:6655643. doi: 10.1155/jonm/6655643 (PMC12714163; doi:10.1155/jonm/6655643)
Supplement: Supplementary file 1 — Supporting Information Additional supporting information can be found online in the Supporting Information section. [file JONM-2025-6655643-s001.docx]

**Supplementary Table 1.** COnsolidated criteria for REporting Qualitative research checklist

Developed from: Tong A, Sainsbury P, Craig J. Consolidated criteria for reporting qualitative research (COREQ): a 32-item checklist for interviews and focus groups. International Journal for Quality in Health Care. 2007. Volume 19, Number 6: pp. 349 – 357.

| **Topic** | **Item No.** | **Guide Questions/Description** | **Reported on**  **Page No.** |
| --- | --- | --- | --- |
| **Domain 1: Research team**  **and reﬂexivity** | | | |
| *Personal characteristics* | | | |
| Interviewer/facilitator | 1 | Which author/s conducted the interview or focus group? | Methods –  Data Collection |
| Credentials | 2 | What were the researcher’s credentials? e.g., PhD, MD | Methods –  Data Collection |
| Occupation | 3 | What was their occupation at the time of the study? | Methods –  Data Collection |
| Gender | 4 | Was the researcher male or female? | Methods –  Data Collection |
| Experience and training | 5 | What experience or training did the researcher have? | Methods –  Data Collection |
| *Relationship with*  *participants* | | | |
| Relationship established | 6 | Was a relationship established prior to study commencement? | Methods –  Data Collection |
| Participant knowledge of  the interviewer | 7 | What did the participants know about the researcher? e.g., personal  goals, reasons for doing the research | Methods –  Data Collection |
| Interviewer characteristics | 8 | What characteristics were reported about the interviewer/facilitator?  e.g., bias, assumptions, reasons and interests in the research topic | Methods –  Data Collection |
| **Domain 2: Study design** | | | |
| *Theoretical framework* | | | |
| Methodological orientation and Theory | 9 | What methodological orientation was stated to underpin the study? e.g., ,grounded theory, discourse analysis, ethnography, phenomenology,  content analysis | Methods – Design |
| *Participant selection* | | | |
| Sampling | 10 | How were participants selected? e.g., purposive, convenience,  consecutive, snowball | Methods – Participants and Setting |
| Method of approach | 11 | How were participants approached? e.g., face-to-face, telephone, mail,  email | Methods – Participants and Setting |
| Sample size | 12 | How many participants were in the study? | Methods – Participants and Setting |
| Non-participation | 13 | How many people refused to participate or dropped out? Reasons? | Methods – Participants and Setting |
| *Setting* | | | |
| Setting of data collection | 14 | Where was the data collected? e.g., home, clinic, workplace | Methods – Participants and Setting |
| Presence of non-  participants | 15 | Was anyone else present besides the participants and researchers? | Methods – Participants and Setting |
| Description of sample | 16 | What are the important characteristics of the sample? e.g., demographic  data, date | Results –  Table 1 |
| *Data collection* | | | |
| Interview guide | 17 | Were questions, prompts, guides provided by the authors? Was it pilot  tested? | Methods – Data Collection and  Supplementary Table 2 |
| Repeat interviews | 18 | Were repeat inter views carried out? If yes, how many? | Methods – Participants and Setting |
| Audio/visual recording | 19 | Did the research use audio or visual recording to collect the data? | Methods –  Data Collection |
| Field notes | 20 | Were field notes made during and/or after the interview or focus group? | Methods –  Data Collection |
| Duration | 21 | What was the duration of the interviews or focus group? | Methods – Data Collection |
| Data saturation | 22 | Was data saturation discussed? | N/A |
| Transcripts returned | 23 | Were transcripts returned to participants for comment and/or correction? | N/A |
| **Domain 3: Analysis**  **and ﬁndings** | | | |
| *Data analysis* | | | |
| Number of data coders | 24 | How many data coders coded the data? | Methods – Data Transfer and Data Analysis |
| Description of the coding  tree | 25 | Did authors provide a description of the coding tree? | N/A |
| Derivation of themes | 26 | Were themes identified in advance or derived from the data? | Methods – Data Transfer and Data Analysis |
| Software | 27 | What software, if applicable, was used to manage the data? | N/A |
| Participant checking | 28 | Did participants provide feedback on the findings? | N/A |
| *Reporting* |  |  |  |
| Quotations presented | 29 | Were participant quotations presented to illustrate the themes/findings?  Was each quotation identified? e.g., participant number | Methods – Data Transfer and Data Analysis |
| Data and findings consistent | 30 | Was there consistency between the data presented and the findings? | Results |
| Clarity of major themes | 31 | Were major themes clearly presented in the findings? | Results –  Table 2 and 3 |
| Clarity of minor themes | 32 | Is there a description of diverse cases or discussion of minor themes? | Results –  Table 2 and 3 |

**Supplementary Table 2.** Guide developed to conduct the focus group

| **Interview phase** | **Target/Function** | **Theme** | ***Question to the focus group*** | ***Follow-up question(s)*** | **Notes** |
| --- | --- | --- | --- | --- | --- |
| *The arrangements should ensure a comfortable conversation atmosphere. Comfortable chairs and the offer of coffee and light pastries increase the comfort feeling* | | | | | |
| Opening | To guarantee comprehensibility and to ensure the course of the interview | Welcome and instructions | *Thank you very much for agreeing to take part in the interview. I shall be recording the conversation so that I can concentrate better on your replies.*  *To be able to take all your remarks into consideration, it is important that you speak one after the other. If necessary, I will remind you about this* | | Ensure a relaxed atmosphere |
| Introduction | Clarify the aims of focus group  Define the characteristics of participants | Shared a common view of moral competent nurse  Collect preliminary data | *A common concept of moral competent nurse was shared*  *Please, fill in the form regarding your age, gender, education, patient association represented, role in the patient association, previous hospitalization experiences* | None | Distribute the short form to collect preliminarily data |
| Key questions | Identify strategies to support the development of a moral competent nurse | Support for morally competent nurses  Tools aimed at supporting the development of a moral competent nurses | *In your opinion, what strategies can support nurses in developing their moral competence?* | *What is required from the:*   - *colleagues?* - *superiors?* - *healthcare organisation?* - *society?* - *other stakeholders?* | Call for brainstorming |
|  |  |  | *What tools can be used in such support according to patients’ representatives?* |  |  |
| Wrapping up | Show appreciation and esteem, clarify outstanding questions | Conclusion | *Before we now come to the end, I would like to know what else you find important about nurses’ moral competence development that was perhaps not (sufficiently) discussed during our talk?* | *What questions have still to be clarified?*  *Would you like to ask me anything else?* | None |
| End | Goodbyes | Thanks | *Then thank you very much, everyone, for the conversation and your frankness as well as the valuable impulses given. I wish you all the best! I’m now switching off the tape.* | |  |
